# Biodistribution and toxicity of pegylated single wall carbon nanotubes in pregnant mice

**DOI:** 10.1186/1743-8977-10-21

**Published:** 2013-06-06

**Authors:** Luisa Campagnolo, Micol Massimiani, Graziana Palmieri, Roberta Bernardini, Cristiano Sacchetti, Antonio Bergamaschi, Lucia Vecchione, Andrea Magrini, Massimo Bottini, Antonio Pietroiusti

**Affiliations:** 1 Department of Biomedicine and Prevention, University of Rome Tor Vergata, Via Montpellier 1, 00133, Rome, Italy; 2 Animal Technology Station, University of Rome Tor Vergata, Via Montpellier 1, 00133, Rome, Italy; 3 Inflammatory and Infectious Disease Center, Sanford Burnham Medical Research Institute, 10901 North Torrey Pines Road, La Jolla, CA 92037, USA; 4 Division of Cellular Biology, La Jolla Institute for Allergy and Immunology, 9420 Athena Circle, La Jolla, CA 92037, USA; 5 Institute of Occupational Medicine, Università Cattolica del Sacro Cuore, Largo F. Vito 1, 00168, Rome, Italy; 6 Department of Experimental Medicine and Surgery, University of Rome Tor Vergata, Via Montpellier 1, 00133, Rome, Italy

## Abstract

**Background:**

Single wall carbon nanotubes (SWCNTs) are considered promising nanoparticles for industrial and biomedical applications; however their potential toxicity in several biological systems, including the feto-placental unit, has been demonstrated. Functionalization of SWCNTs with polyethylene glycol chains (PEG-SWCNTs) dramatically reduces their toxicity, and for this reason PEG-SWCNTs are candidates for biomedical applications. However, no data are available on their safety for the developing embryo, in spite of the clinical and social relevance of this topic. The purpose of this study is therefore to investigate the safety of PEG-SWCNTs for their use as biomedical carriers in pregnancy.

**Methods:**

For toxicological studies, amino-functionalized PEG-SWCNT were intravenously injected in CD1 pregnant mice at different doses (range 0.1-30 μg/mouse), in single or multiple administrations. For biodistribution studies, fluorescently labeled PEG-SWCNTs were obtained by acylation of terminal PEG amino groups with near infrared emitting fluorochromes (PEG-SWCNT-750) and injected at the dosage of 10 μg/mouse, at either day 5.5 (when the placenta is still developing) or day 14.5 of gestation (when the maturation of the placenta is complete).

**Results:**

We found no adverse effects both on embryos and dams up to the dose of 10 μg/mouse. At the dose of 30 μg/mouse, occasional teratogenic effects, associated with placental damage, were detected both when administered as a single bolus (1 out of 10 dams; 1 malformed embryo) or as multiple doses (2 out of 10 dams; 5 malformed embryos). The difference in the prevalence of dams with malformed embryos between the 30 μg exposed group and controls approached the statistical significance (p = 0.06). Hepatic damage in dams was seen only in the multiple exposure group (4 out of 10; p = 0.04 when compared with the single exposure group or controls). PEG-SWCNT-750 reached the conceptus when administered early in pregnancy. At later stages, PEG-SWCNT-750 were detected in the placenta and the yolk sac, but not in the embryo.

**Conclusions:**

PEG-SWCNTs may cause occasional teratogenic effects in mice beyond a threshold dose. Such effect might depend on their ability to reach the feto-placenta unit. Although not automatically transferable to humans, these data should be considered if exposing women during pregnancy.

## Introduction

Since their discovery, almost 20 years ago, carbon nanotubes (CNTs), a class of fiber-shaped nanoparticles (NPs), have been indicated as good candidates for many applications in industrial and biomedical settings. For such reason, their biocompatibility has been extensively investigated over the last 10 years, and evidences that CNTs might have negative effects in biological systems have been reported both *in vitro* and *in vivo*, as recently reviewed by Shvedova et al. [1]. The main targets of occupational and environmental exposure to CNTs are the lungs, the skin and the gastro-intestinal tract. Due to their small size (at least one dimension less than 100 nm), nanoparticles might cross biological barriers [2], and CNTs have been demonstrated to reach the brain after being orally administered, indicating that they can access protected niches [3]. The finding of serious embryotoxic effects after single wall CNT (SWCNT) administration to pregnant mice suggests that the placenta might be crossed by these NPs [4], although nanoparticles might also induce toxicity by locally interfering with placental functions. Studies specifically addressing this issue have not yet been performed.

In addition to the unintended occupational and environmental exposure, people may be purposefully exposed to CNTs, given the ability of this material to carry diagnostic and therapeutic probes with high efficiency [5-10]. For biomedical uses, CNTs are generally chemically modified (functionalized) in order to improve some physical properties, such as solubility in organic or aqueous solvents and biological fluids. One of the most used methods of functionalization is represented by conjugation with polyethylene glycol (PEG) [11], and the absence of acute and chronic adverse effects after intravenous administration of single wall CNTs (SWCNTs) conjugated with PEG (PEG-SWCNTs) in mice has been reported [12]. However, no data are currently available on the effects of PEG-SWCNT exposure during pregnancy in dams and embryos. This matter is relevant, not only for the obvious clinical and social implications, but also in light of the high sensitivity of embryonic tissues to the toxic effects of CNTs, which induce severe embryo abnormalities at doses having no toxic effects on maternal organs [4].

**Figure 1:**
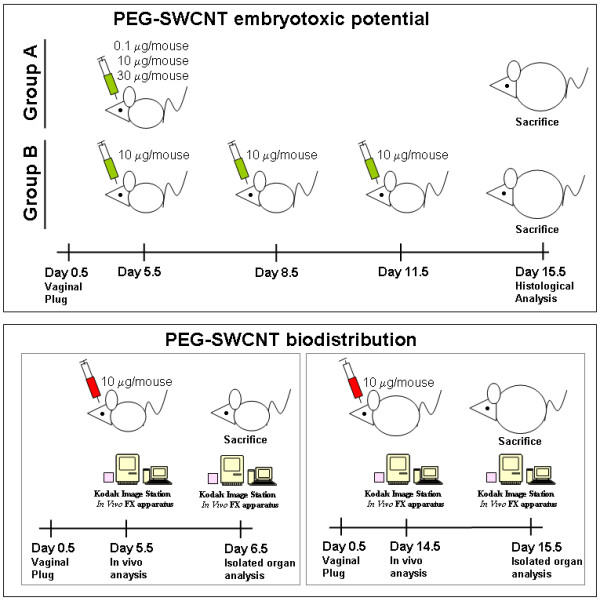
**Schematic representation of the experimental design for the evaluation of PEG-SWCNT embryotoxic potential and biodistribution.** Upper panel: Animals were divided into two groups, **A** and **B**. Group A received a single administration of either 0.1 (5 females), 10 (10 females) or 30 (10 females) μg/mouse PEG-SWCNTs or PBS (18 females), at 5.5 dpc; group B received three administrations of 10 μg/mouse PEG-SWCNTs (10 females) or PBS (10 females), at different times of gestation. Animals were sacrificed at day 15.5 of gestation and maternal tissues, placentas and fetuses were analyzed. Lower panels: For biodistribution studies, PEG-SWCNT-750 were administered either at 5.5 dpc (5 females) or 14.5 dpc (5 females) and isolated organs studied either at 6.5 dpc or 15.5 dpc, respectively.

In this study, we assessed the possible maternal and fetal effects of PEG-SWCNTs when administered to pregnant female mice as a bolus or after repeated administrations (Figure 1). In order to clarify if SWCNTs may cause embryotoxic effects by directly reaching the feto-placental unit, or indirectly through mother-mediated mechanisms, we performed biodistribution studies in early pregnancy, when the placenta is shaping, and at a later stage, characterized by the presence of a fully developed placenta (Figure 1).

## Results and discussion

### Production and characterization of PEG-SWCNTs

**Figure 2:**
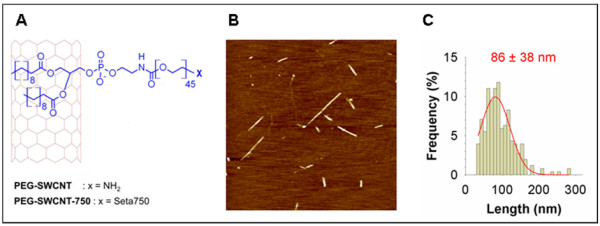
**Production and characterization of PEG-SWCNTs. A**. PEG-SWCNT were fabricated by the adsorption onto pristine SWCNTs of phospholipids modified with PEG chains carrying an amino group at their end. PEG-SWCNT-750 were obtained by capping the PEG chain terminal amino groups on PEG-SWCNTs with NIR-emitting fluorochrome (Seta750). **B**. AAC-mode AFM image of PEG-SWCNTs deposited on freshly cleaved mica sample (scan size 2.5 × 2.5 μm). **C**. PEG-SWCNT length distribution (N = 256).

Individual (non-aggregated) amino-functionalized PEG-SWCNTs were fabricated from the processing of commercially available single SWCNTs through a non-covalent protocol based on the adsorption onto SWCNT sidewalls of phospholipids modified with PEG chains, carrying amino groups at their end. Phospholipids bind strongly to SWCNTs through hydrophobic interactions between the fatty acid aliphatic chains and the graphitic sidewalls, leaving the hydrophilic PEG chains protruding from the sidewall [13]. PEG chains improve the steric stability of the nanoparticles and allow further functionalization [13]. Fluorescent PEG-SWCNTs (PEG-SWCNT-750) were obtained by capping the PEG chain terminal amino groups on PEG-SWCNTs with near infrared (NIR)-emitting fluorochromes (Figure 2A).

PEG-SWCNTs and PEG-SWCNT-Seta750 were dispersed in PBS (pH 7.4) and formed a stable dispersion of individual nanoparticles upon standing for several months at room temperature [14]. MALDI and elemental analysis data confirmed the presence of intact 2 kDa-mw PEG chains decorating nanotube sidewalls and the absence of impurities, respectively (data not shown). AFM images showed the presence of individual particles composed by short PEG-SWCNTs having a narrow distribution of lengths centered at approximately 90 nm (Figure 2B and C). Finally, we calculated by using our published model [15] that the PEG density was about 0.1 mmol per gram of nanotube material, corresponding to approximately 15 PEG chains per nanotube. Analysis of possible endotoxin contamination of nanoparticle suspensions revealed a content of 3 × 10^-3^ EU/ml, that was highly below the FDA limit of 0.5 EU/ml (not shown).

### *In vivo* effect of PEG-SWCNTs

To investigate possible embryotoxicity of PEGylated nanotubes, different doses were intra-venously administered to pregnant mouse females, either as a single bolus (range 0.1-30 μg/mouse) or in multiple administrations (10 μg/mouse each). The intra-venous route was chosen since PEG-SWCNTs have been developed as putative molecular carriers for biomedical purposes. For females undergoing a single injection, nanoparticles were administered at day 5.5 of gestation (5.5 dpc, group A in Figure 1), when embryos have just implanted, and the definitive placenta is not yet formed, so that the embryo is separated from the uterine tissue by a layer of trophoblast giant cells and extra-embryonic tissues. Dams were exposed to 0.1 (5 mice), 10 or 30 μg/mouse (10 mice each) (Figure 1; group A). For multiple exposures, females received 3 refracted doses of 10 μg/mouse each (10 females; group B in Figure 1) and injections were performed every three other days, starting from 5.5 dpc. For this group, exposure to PEG-SWCNTs occurred during different stages of placental development. The presence of placental and/or fetal abnormalities was assessed in all groups at day 15.5 of gestation. In parallel, some key maternal organs, such as liver, lungs, heart, spleen and kidneys were collected for histological examination.

Females receiving up to 10 μg/mouse of PEG-SWCNTs did not carry any abnormal fetus nor displayed tissue histological alterations. By contrast, at the highest concentration tested we observed fetal abnormalities in one in ten dams (1/10) exposed to a single injection of 30 μg/mouse (1 malformed embryo) and in 2 out of 10 dams exposed to the three refracted doses of 10 μg/mouse each (5 malformed embryos). In both groups similar fetal malformations were observed, consisting in delayed development of the paws and head abnormalities. In 4 fetuses out of 6, these malformations were associated with reduced length (around 1.2-1.3 cm), which was out of two standard deviations in comparison to our internal growth reference curves. The main results of these experiments are summarized in Table 1.

**Table 1:** Summary of the main parameters evaluated for the embryotoxicity studies

	**♀**	**Mean No. fetuses/female**	**CRL**	**Fetal weight (mg)**	♀** with malformed fetuses**	**Total No. of resorptions**
CTRL	18	13.7 ± 1.3	1.57 ± 0.15	401 ± 11	0	14
100 ng/mouse	5	12.3 ± 1.3	1.60 ± 0.21	396 ± 9	0	3
10 μg/mouse	10	14.0 ± 0.4	1.57 ± 0.14	412 ± 3	0	7
30 μg/mouse	10	12.9 ± 0.7	1.52 ± 0.12	399 ± 14	1/10 (1 fetus)	8
CTRL 3 × 10	10	12.2 ± 1.0	1.61 ± 0.11	392 ± 15	0	8
3 × 10 μg/mouse	10	12.7 ± 1.8	1.58 ± 0.10	395 ± 17	2/10 (3 + 2 fetuses)	7

♀: number of females analyzed.

*CRL*: crown rump length.

Although no statistically significant difference or trend was observed when comparing mice exposed to 30 μg as a single dose with those exposed three times to 10 μg, a trend toward significance was seen when comparing this latter group with controls (p = 0.11). Furthermore, when grouping animals exposed to 30 μg/mouse (as single or repeated administrations), the difference with controls approached the statistical significance (p = 0.06).

From a clinical point of view, such results suggest that the fractionated administration of a dose causing embryotoxicity, when given as a bolus, does not decrease the toxic potential, but may actually increase it, presumably as a consequence of nanoparticle accumulation in tissues, and/or exposure during different stages of pregnancy, when the placental barrier displays different permeability [16]. In this respect, the evaluation of repeated exposures is particularly relevant in pregnancy, due to the dynamic nature of this physiologic process during which both the sensitivity of the embryo to xenobiotics, and the efficiency of the placenta as a biological barrier change depending on the developmental stage.

**Figure 3:**
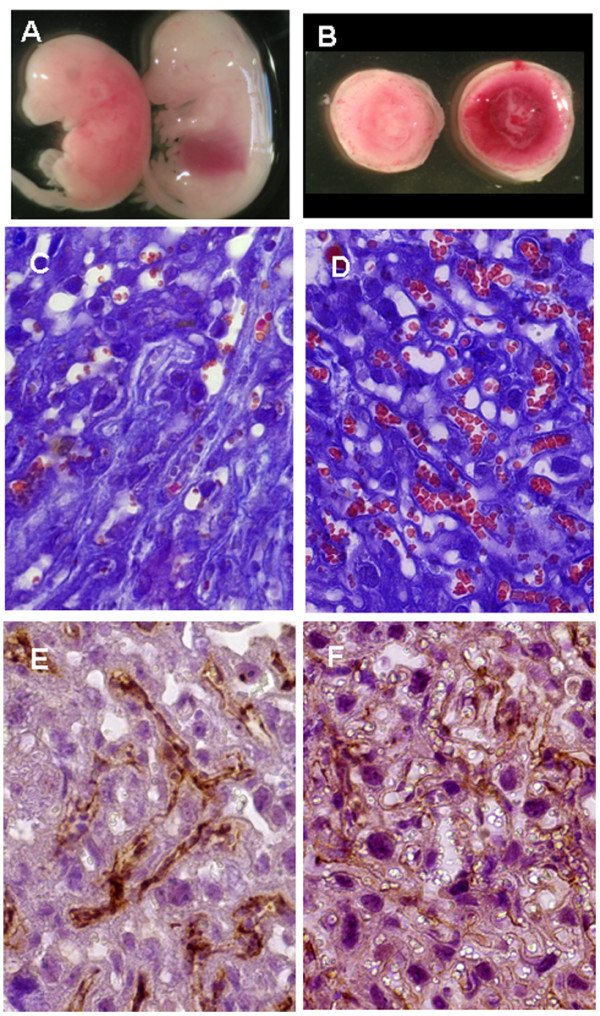
**Macroscopic and microscopic analysis of placentas and embryos after maternal exposure to PEG-SWCNTs. A**. A Fetus showing abnormal development (left) is compared to one from a control female (right). **B**. The placenta nourishing the abnormal fetus (left) appeared abnormally vascularised and slightly smaller than the one from the control fetus (right). **C-F**. Reduced vascularisation in abnormal placentas (**C**) compared to normal ones (**D**) was demonstrated in Azan-Mallory stained sections; similar results were obtained by immunostaining, using the anti-CD31 antibody: (**E**) placentas nourishing abnormal fetuses displayed reduced CD31 expression, compared to normal tissue (**F**).

Fetal abnormalities were always associated with abnormal placental development (Figure 3A and B) consisting in a moderate reduction in the size (Figure 3B), accompanied by a dramatic reduction in the vascularization of the labyrinth layer (Figure 3C and D) and in the expression of the pan-endothelial marker CD31 (Figure 3E and F), thus indicating altered development and vascularization; similar phenotype has been described in several mouse mutant models (e.g., notch signalling pathway mutants) [17-19], and in placentas after the administration of non pegylated SWCNTs and silica and titania nanoparticles [4,20] suggesting that administration of nanoparticles early in gestation might interfere with mechanisms important for placental morphogenesis.

**Figure 4:**
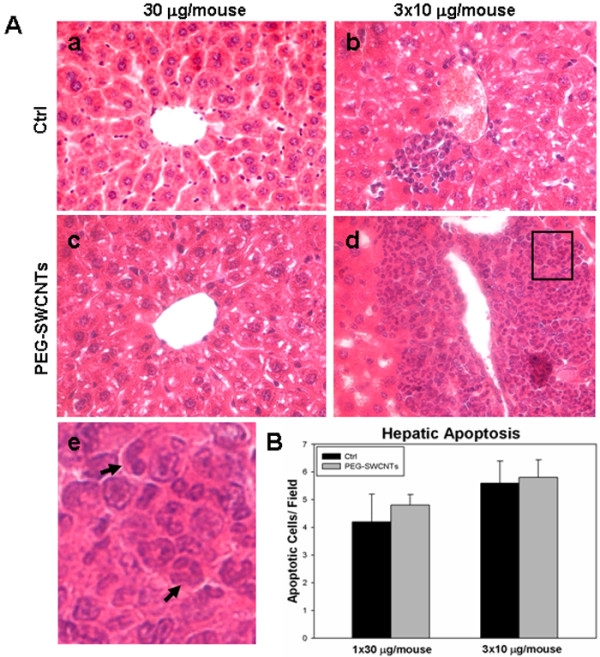
**Histological analysis of maternal liver after exposure to PEG-SWCNTs. A**. Liver sections from dams exposed to vehicle only (**a**, **b**), 30 μg (**c**) or to three repeated administrations of 10 μg each (**d**) were stained with Hematoxylin and Eosin. In the liver from animals that received three administrations, areas of extramedullary hematopoiesis were in some cases evidenced (**d**). (**e**) Magnification of the boxed area in d shows the presence of monocyte and granulocyte like cells (arrows). **B.** Histograms show no differences in the number of apoptotic cells in all liver samples analyzed.

**Figure 5:**
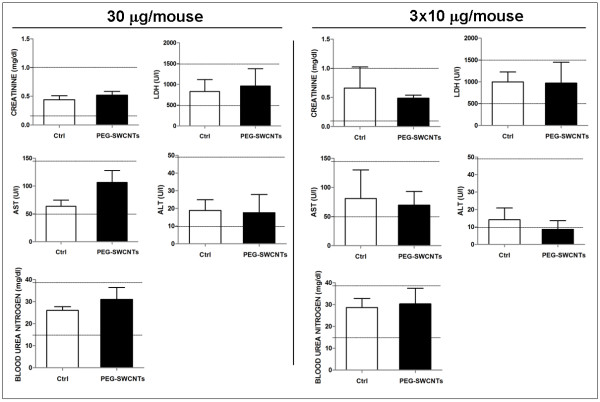
**Determination of biochemical parameters in maternal blood.** Values (mean ± SD) measured in the serum of pregnant females exposed to either 30 μg/mouse in a single administration or in three repeated administrations of 10 μg each. Aspartate and alanine aminotransferase (AST and ALT), blood urea nitrogen, creatinine and lactate dehydrogenase (LDH) were evaluated. Reference ranges reported as horizontal lines, were obtained from mice of the same strain, sex, and age.

With respect to the organs of dams exposed to a single administration of PEG-SWCNTs, at all tested doses, no evident structural alterations were identified in lung, heart, kidney, spleen and liver (not shown). At contrast, females undergoing multiple administrations displayed a non significant increase in spleen weight compared to controls (0.36 ± 0.04 gr *vs* 0.33 ± 0.03 gr), and liver alterations characterized by clusters of granulocytes, monocytes and megacaryocytes (Figure 4A, b, d and e), suggesting the occurrence of extramedullary myelopoiesis in the liver (4 out of 10 females; p = 0.04 when compared with the single exposure group). No similar phenotype was ever observed in control animals. Since extramedullary myelopoiesis is observed during immune responses and inflammation [21], it is reasonable to hypothesize that the repeated administration of PEG-SWCNTs induced a hepatic inflammatory response. This observation is in line with previous data from the literature indicating that the liver is one of the main organs of nanoparticle accumulation and damage [22,23]. To further investigate hepatic damage, we evaluated the presence of apoptotic cells in liver tissues: no sign of apoptosis was detected in livers collected from pregnant females exposed to either 30 μg in a single administration or to the three repeated administrations of 10 μg each (Figure 4B). Similarly, in the two groups, no alterations of biochemical parameters, such as liver enzymes aspartate aminotransferase and alanine aminotransferase, blood urea nitrogen, creatinine and lactate dehydrogenase were recorded (Figure 5).

No other significant differences were observed with respect to the weight of dams and normal fetuses, the number of pups per litter and the number of resorptions observed in control and treated females (Table 1). In details, the maternal body weight gain was almost identical in exposed and non exposed dams, being less than 1% from gestational day 5.5 to gestational day 8.5; from day 9.5 of pregnancy to 11.5 it approached 10%. On day 12.5 it reached 20% and subsequently, up to 15.5 (the day of sacrifice) a daily 10% increase was observed. From day 8.5 to day 15.5 the difference in weight gain between exposed and non exposed dams was always less than 1%.

Altogether, these observations suggest that exposure to PEG-SWCNTs is in general well tolerated by adult tissues, although a mild inflammatory response in the liver might be observed after repeated administrations. These results were further confirmed by the absence of any clinical alterations in the exposed animals compared to controls, such as food and water consumption, weight gain and behaviour.

### Biodistribution analysis of fluorescently labeled PEG-SWCNTs

**Figure 6:**
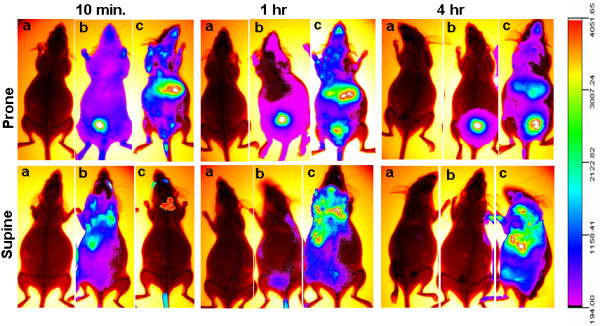
***In vivo *analysis of the biodistribution of fluorescently labelled PEG-SWCNTs.** Panels show recording of fluorescence in mice injected with PBS (**a**) Seta750 (**b**) and PEG-SWCNT-750 (**c**). No fluorescence was recorded in control mice (**a**) at any time. Already 10 min after the injection, animals that received the fluorophore Seta750 alone (**b**) displayed fluorescence almost exclusively localized in the bladder. High fluorescence was recorded in liver and brown fat of mice administered with PEG-SWCNT-750 (**c**); one hour after the injection bladder accumulation of fluorescence was also visible in this group.

**Figure 7:**
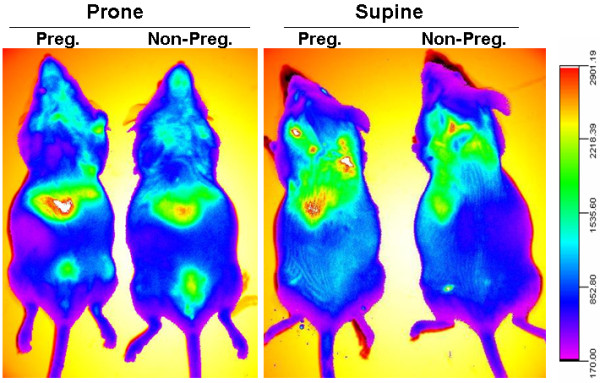
**Comparison of *in vivo *biodistribution of PEG-SWCNT-750 in pregnant and non-pregnant animals.** Recording of fluorescence shows an almost identical distribution pattern of PEG-SWCNT-750 in pregnant and non pregnant mice.

**Figure 8:**
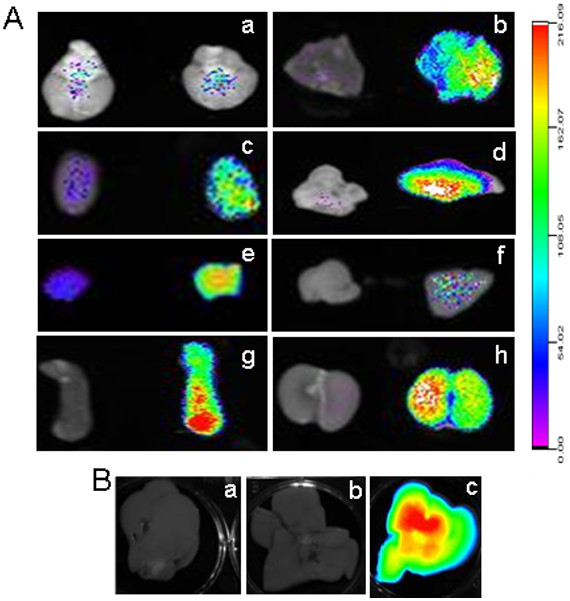
**Biodistribution of PEG-SWCNT-750 in isolated maternal organs.** 24 hrs after the injection, maternal organs were collected and fluorescence recorded. **A**: with the exception of the brain (**a**), all organs from animals injected with PEG-SWCNT-750 (right column) displayed much higher fluorescence than the controls (left column). **b**: lung; **c**: heart; **d**: brown fat; **e**: thymus; **f**: pancreas; **g**: spleen; **h**: kidney. **B**: comparison of fluorescence distribution in liver from dams that were exposed to PBS (**a**), Seta750 (**b**) or PEG-SWCNT-750 (**c**).

In order to assess if the particles were able to reach the placenta and the embryo, 5.5 day pregnant females were injected with either 10 μg/mouse of fluorescently labeled PEG-SWCNTs (PEG-SWCNT-750), with the unconjugated fluorophore (Seta750), or with the vehicle (PBS). Localization of fluorescence in tissues was studied using the Kodak Image Station *In Vivo* FX apparatus. As expected, *in vivo*, a bright fluorescence was localized to the liver of mice injected with PEG-SWCNT-750 (Figure 6c, upper panel), due to the role of this organ in cleansing the blood from xenobiotics, including nanoparticles [24-26]. In addition, bright fluorescence was registered in the neck region, compatible with the localization of the highly vascularised brown fat (Figure 6c, upper panel). To rule out the possibility that the visualized fluorescence might be consequence of fluorophore detachment, a group of pregnant females was injected with the Seta750 fluorophore itself. The body clearance of the fluorophore was very rapid, and already 10 minutes after the injection, accumulation was visible mainly in the bladder, reflecting fast renal excretion (Figure 6b, upper panel). Bladder fluorescence was also detected in animals exposed to PEG-SWCNT-750, but only after one hour from the injection, indicating that also in this case renal excretion was occurring, although with a slower rate; in these females, fluorescence distribution was substantially unchanged up to 4 hrs, while in the group that received the unconjugated fluorophore after 4 hours fluorescence was localized exclusively to the bladder. Due to the site of injection, animals often displayed fluorescence in the eyes. Interestingly, comparison between body distribution of both PEG-SWCNT-750 and Seta750 in non-pregnant and pregnant animals showed no substantial differences, indicating that pregnancy *per se* does not alter nanoparticle body distribution (Figure 7). After 24 h from the injection, animals were sacrificed and maternal organs, placentas and fetuses collected for fluorescence evaluation. Fluorescence was observed in all organs isolated from the PEG-SWCNT-750 treated females (Figure 8), with the exception of the brain (Figure 8A, a), suggesting that these nanoparticles do not cross the blood brain barrier, or do that in very little amount, below the detection limit of our system that is around 0.2-0.5 nM (corresponding to about 25-50 ng/ml). No fluorescence was detectable in any of the organs isolated from the Seta750 treated females, further supporting the fast renal excretion of the non-conjugated fluorophore; a representative image of the liver is shown in Figure 8B. Taken together these results indicate that the fluorescence detected in the PEG-SWCNT-750 treated animals reflected the presence of the nanoparticles.

**Figure 9:**
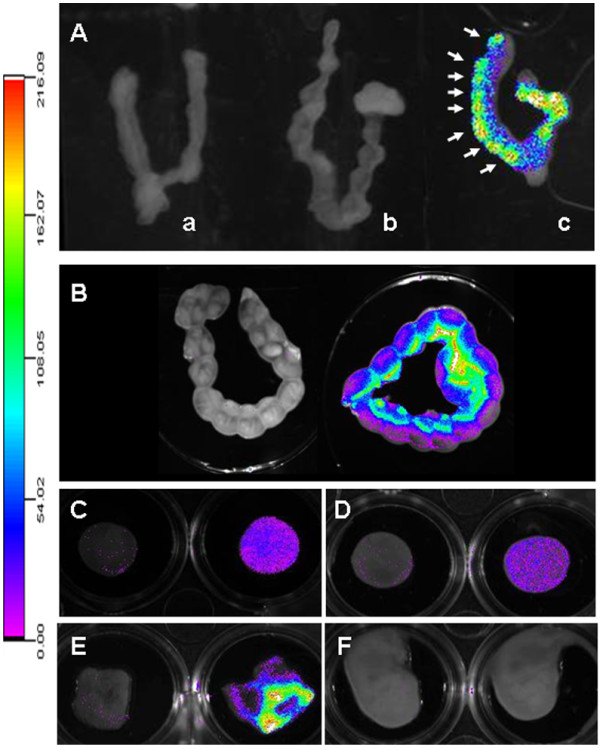
**Analysis of the biodistribution of PEG-SWCNT-750 in the uterus, placenta, fetus and fetal membranes. A**: at 6.5 dpc isolated uteri from dams exposed at 5.5 dpc to PEG-SWCNT-750 showed fluorescence in correspondence of the implanted embryos (**c**, arrows). No fluorescence was recorded in uteri collected from females exposed to either PBS or Seta750 (**a** and **b**, respectively). **B**: fluorescence recorded in uteri collected from dams at 15.5 dpc that were either exposed to PBS (left) or PEG-SWCNT-750 (right) at day 14.5 of gestation. C and D: 15.5 dpc isolated placentas from mice administered with PBS (left) or PEG-SWCNT-750 (right) with maternal (**C**) or fetal (**D**) side facing up. E and F: 15.5 dpc fetal membranes (**E**) and fetuses (**F**) from PBS (left) or PEG-SWCNT-750 (right) treated dams.

With respect to the distribution of PEG-SWCNT-750 to the implantation sites and its localization in the placenta and the embryo, we observed that, when administered early in gestation (5.5 dpc), the particles reached the uterus in detectable amounts and localized in correspondence of every implantation site (Figure 9A arrows). In contrast, uteri from Seta750 treated females did not show any fluorescence. Similar results were obtained when PEG-SWCNT-750 were administered at midgestation (14.5 dpc), when the placenta has been already completely formed (Figure 9B). Fluorescence localized to every implantation site, with a brighter signal corresponding to the mesometrial side of the uterus, where the placenta is located. Pregnant uteri were dissected, and isolated placentas and fetuses analyzed for fluorescence (Figure 9C-F). Interestingly, we could detect PEG-SWCNT-750 in all the placentas, both on the maternal and fetal side (Figure 9C and D), but not in fetuses (Figure 9F). A bright fluorescence was also registered in the yolk sac (Figure 9E). This observation is in line with published data showing that gold nanoparticles may cross both the chorioallantoic placenta and the yolk sac placenta [27].

In spite of a possible transplacental crossing, we did not detect fluorescence in the fetus, probably as a consequence of a too high dilution of the particles, below the detection limit of our system. On the other hand, given the relatively low concentrations of PEG-SWCNTs used in the present study, the possibility to identify the very low fraction possibly crossing the placenta by Transmission Electron Microscopy is very unlikely, also in consideration of the short length, carbonaceous nature and lack of aggregates. However, it is possible that accumulation of nanoparticles in the placenta might indirectly induce embryonic damage, independently from transplacental crossing. In support to this hypothesis, using an *in vitro* placental model, it has been recently shown that contact of nanoparticles with trophoblast cells caused alteration in the underlying embryonic cells, in the absence of transplacental passage [28].

Although our data refer to mouse placenta, there is strong suggestion that nanoparticles may cross human placenta also. In fact, in a human *ex vivo* model, Wick et al. [29] clearly showed transplacental passage of polystirene nanoparticles. This experiment has however time limitations (it may last only few hours) and may be performed only in placentas at term; therefore no information may be obtained, for examples, on transplacental crossing during the very early stages of development of this organ.

## Conclusion

Our data indicate that PEG-SWCNTs might have a mild embryotoxic effect in exposed mice, since relatively high doses may cause occasional teratogenic effects after maternal intravenous exposure. We found embryotoxicity of PEG-modified carbon nanotubes at a dose of 30 μg/mouse. This dose is equivalent to an approximate 1.2 mg/kg dose (considering a mouse weight of 25 g) or an approximate 70 mg dose for a 60 kg pregnant patient. It is reasonable to suppose that such dose might be used for biomedical application of PEG-modified carbon nanotubes in humans.

Due to the interspecies differences, the question if these nanotoxicological data can be related to humans arises. The mouse placenta is structurally different from the human placenta, however considerable similarities exist between the two species [30]: both placentas are defined as emochorial, with the maternal blood directly coming in contact with a layer of syncytiotrophoblast lining the fetal villi, and placental permeability to some molecules is similar in both species [31]. In addition, molecular pathways of mouse placental development are conserved in humans [30]. These observations suggest that the mouse can be considered a good model to obtain indications on the teratogenic potential of nanoparticles, although further studies using human models (e.g., the *ex vivo* human placenta model or *in vitro* systems) are desirable.

An interesting finding of this study is that exposure to fractionated doses, given at different stages of pregnancy, cause mild liver damage in dams and perhaps increase the teratogenic effects. However, given the relatively limited size of the exposed female population, further confirmatory studies are needed to clarify the issue

We also demonstrated that PEG-SWCNTs are able to reach and damage the placenta, an event associated with alteration of fetal development. In our experimental model, we could not detect PEG-SWCNTs in fetal tissues; however evidence of their presence in the fetal compartment of the placenta and the yolk sac suggests that a contact with the fetus might occur under appropriate circumstances.

In light of all the above reported considerations, PEG-SWCNTs should be used with caution as nanodelivery system during pregnancy.

## Methods

### Production of PEG-SWCNTs

Commercially available SWCNTs were purchased from Carbon Solution Inc. (Riverside, CA). They were purified by air oxidation. Amino-functionalized PEGylated SWCNTs (PEG-SWCNT) were fabricated through our published non-covalent protocol [15] based on the adsorption of PEG-modified phospholipids onto SWCNTs’ sidewalls. Briefly, 5 mg of pristine (non-modified) SWCNTs (Carbon Solutions, Riverside, CA) were oven dried at 160°C for 3 h, sonicated with 25 mg of phospholipids modified with amino-functionalized 2-kDa molecular weight (mw) linear PEG chains (DSPE-PEG(2 k)-NH_2_, Avanti Polar Lipids, Alabaster, AL) in PBS for 6 h in an ultrasonic bath (Ultrasonic Cleaner, Cole-Parmer, Vernon Hills, IL) and ultracentrifuged (Optima™ XL-80 K Ultracentrifuge, Beckman, Palo Alto, CA) at 20,000 × *g* for 6 h at 4°C. Approximately 80% of the supernatant fraction was collected and ultracentrifuged at 40,000 × *g* for 6 h at 4°C. Approximately 80% of this second supernatant fraction was collected and ultracentrifuged at 70,000 × *g* for 6 h at 4°C. The resulting pellet was dispersed in PBS and filtered through 100 kDa mw-cut off centrifugal devices (Vivaspin 500, Sartorius, Göttingen, Germany) to remove free phospholipids. All the fabrication steps were carried out under conditions of sterility. To carry out investigations about the accumulation profile of PEG-SWCNTs in living animals, fluorescent PEG-SWCNTs (PEG-SWCNT-750) were obtained by acylation of terminal PEG amino groups with *N*-hydroxysuccinimide ester (NHS)-modified near infrared (NIR)-emitting fluorochromes (Seta750-NHS, SETA BioMedicals, Urbana, IL). The fluorochrome had excitation and emission distribution maxima at 751 nm and 779 nm (in PBS, pH 7.4), respectively, and it was chosen in order to minimize the background noise arising from tissue auto-fluorescence.

PEG-SWCNTs were characterized through elemental analysis, atomic force microscopy (AFM) and matrix-assisted laser desorption/ionization (MALDI). Elemental analysis was performed on a samples containing 100 μg/ml of nanoparticles by Elemental Analysis Inc. (Lexington, KY) utilizing Proton Induced X-ray Emission (PIXE). In PEG-SWCNT samples the amount of metallic contaminants was under the detection limit of the technique (0.001 μg/cm^2^). Samples for AFM imaging were prepared as follow: first 10 μL of PEG-SWCNTs in PBS (concentration 2.5 μg/ml) were dropped onto a freshly cleaved mica substrate (Ted Pella, Redding, CA), next the droplet was allowed to stand for a couple of minutes at room temperature, and finally the mica surface was rinsed with water and dried under a gentle nitrogen stream. AFM images were recorded using a 5500 AFM (Agilent Technologies, Santa Clara, CA) in acoustic alternate current (AAC) mode. MALDI samples were prepared as follow: 1 μL of PEG-SWCNTs in PBS diluted 1:1 with matrix solution [10 mg/mL α-cyano-4-hydroxycinnamic acid in 50% (v/v) acetonitrile/0.1% (v/v) trifluoroacetic acid/50% (v/v) water] was dropped onto MALDI plate and allowed to dry at room temperature. MALDI spectra were recorded by means of an Autoflex™ II TOF/TOF (Bruker Daltonics Inc., Billerica, MA).

### Dispersion and stability of PEG-SWCNTs

PEG-SWCNTs and PEG-SWCNT-Seta750 were dispersed in PBS (pH7.4) at room temperature (approximately 23°C). Stability of the suspensions was investigated by recording the UV–vis absorbance spectrum at various storage times. Both PEG-SWCNT and PEG-SWCNT-Seta750 freshly fabricated dispersions (150 μg/ml) showed UV–vis absorbance spectra characterized by distinct and sharp peaks corresponding to the van Hove singularity transitions; thus suggesting the presence of individually dispersed nanoparticles [14]. The UV–vis spectra did not display any changes upon storing nanotube solutions for several months at room temperature in PBS, thus suggesting absence of aggregation.

For animal exposure experiments, PEG-SWCNT solutions that were not older than 1 month were used. The UV–vis absorbance spectrum of nanotube solutions was regularly checked before their use in order to confirm that they were formed by individual (not-aggregated) nanoparticles. Stability of the fluorescent tag (Seta750) conjugated to PEG-SWCNT-750 was also investigated. PEG-SWCNT-750 solution was stored for two weeks at room temperature in PBS, filtered once through 100 kDa-mw cut-off centrifugal filtering devices and the UV–vis absorbance spectrum of the eluate recorded. The latter showed a very faint peak centred at approximately 750 nm, thus suggesting that the release of the fluorescent tag from PEG-SWCNT-750 was minimal. For the determination of endotoxin content of PEG-SWCNT samples, the LAL Chromogenic Endotoxin Quantitation kit (Thermo Scientific, Rockford, IL, USA) was used.

### Animal model

In the present study pregnant and non-pregnant females of the CD1 strain were used; such outbred strain is considered a multipurpose model suitable for toxicological studies. Animals were housed and mated under standard laboratory conditions and treated using humane care in order to inflict the least possible pain. All experiments were approved by the Institutional Animal Care and Use Committee (IACUC) and carried out according to the Italian and European rules (D.L.vo 116/92; C.E. 609/86; European Directive 2010/63/EU). A veterinary surgeon has been present during the injection and blood sample collection experiments. Animal handling, before and after experiment, has been carried out only by trained personnel.

### *In vivo* analysis of fluorescently labeled PEGylated-SWCNT biodistribution

For the evaluation of nanoparticle distribution in maternal organs, placentas and fetuses, mice were administered with fluorescently labeled PEG-SWCNTs (PEG-SWCNT-750; 5 animals), or with the fluorochrome Seta750 only (5 females), at the same concentration as that conjugated to the nanotubes. The concentration of free Seta 750 in PBS was calculated by recording the absorbance spectrum of Seta750 in PBS and dividing the value of absorbance at 750 nm by the extinction coefficient furnished by the supplier. The concentration of Seta750 bound to the nanotubes was calculated by a two-step process. First, the absorbance spectrum of Seta750 bound to the nanotubes was calculated by subtracting the absorbance spectrum of fluorochrome-devoid nanotubes from the spectrum of nanotubes loaded with fluorochromes. Next, the obtained value of absorbance at 750 nm was divided by the extinction coefficient furnished by the supplier. Control animals (5 animals) received the same volume of the dispersant medium (PBS). Animals were intra-venously injected with 10 μg of nanoparticles in a volume of 100 μl at either day 5.5 or day 14.5 of gestation, via the retro-bulbar plexus, as previously reported [4]. Such route of administration is considered an alternative to the tail vein injection and is recommended for small laboratory animals [32], since it is much less technically challenging, does not require warming preparation of the animal or anesthesia, and the whole procedure is only a matter of seconds. For nanoparticle administration, the mouse was immobilized on absorbent paper, keeping it motionless, and a volume of 100 μl was gently injected in the center of the retro-orbital sinus of the right eye, by using a 1 cc syringe equipped with a 27 gauge needle. No local complications related to the procedure, such as local edema or relevant bleeding, were ever observed. Biodistribution of fluorescence was analyzed using a Kodak Image Station *In Vivo* FX apparatus after 10 minutes (time 0), 1 hour (time 1) and 4 hours (time 2). In order to determine the detection limit of our system, we have evaluated fluorescence of PEG-SWCNT-750 samples of scalar concentrations and observed no fluorescence between 25 and 50 ng/ml. For comparing tissue distribution between pregnant and non pregnant animals, non-pregnant females were also exposed to nanoparticles. Recording of the fluorescence was obtained during anesthesia, (Avertin 250 mg/kg) with the animals lying in the prone and supine position. Anesthesia lasted up to about 4 hr, that was the duration of fluorescence recording in live animals. After recovering from anesthesia, animals were placed back in their cages. After 24 hr, animals were euthanized and immediately placed in the Kodak apparatus for further evaluation of fluorescence distribution. Maternal organs, placentas and fetuses were then isolated and their fluorescence recorded.

### Evaluation of PEG-SWCNT embryotoxic potential

PEGylate-SWCNTs were intravenously administered to pregnant females as above reported. Briefly, six to eight week old CD1 females were used. The mean age in all groups ranged from 6.8 to 7.2 weeks. For mating, 2-3 females were distributed in each cage containing one male of proven fertility. For each experiment, 5 females were randomly allocated to the different exposure groups, each of which had a predetermined final size of at least 5 animals. The presence of a vaginal plug was checked every morning, and the day of the vaginal plug was considered day 0.5 of gestation.

Pregnant females were divided in two groups, depending on the type of analysis intended: group A received either PEGylate-SWCNTs or vehicle on day 5.5 of gestation (5.5 dpc). For this group, administered doses were either 0.1 (5 females), 10 (10 females) or 30 μg/mouse (10 females, Table 1 and Figure 1). Group B was administered with a total amount of nanoparticles of 30 μg/mouse (10 females), but in three refracted doses of 10 μg/mouse, on day 5.5, 8.5 and 11.5 of gestation. In this case, intra venous administration was performed alternating injections in the right and left retro-orbital plexus, so that administration through the same eye occurred after six days. Control animals (18 for single administration and 10 for repeated injections) were administered with the PBS, that was the medium in which nanoparticles were dispersed. No local complications secondary to the injection procedure were observed.

The doses of PEG-SWCNTs used in this study are in the lower range of those employed in *in vivo* studies, showing no toxic effect in adult animals [12].

All groups were sacrificed at 15.5 dpc using carbon dioxide, and their organs, placentas and fetuses collected for further analyses. Maternal organs from all animals, including liver, lung, kidney and spleen were fixed and processed for paraffin embedding. Spleens were weighted before fixation. Placentas and fetuses were carefully evaluated for the presence of malformations under a stereomicroscope. Fetuses that presented evident morphological abnormalities were photographed and then fixed with their placentas in 4% paraformaldehyde together with a morphologically normal sibling for subsequent histochemical and immuno-histochemical analysis. In parallel, fetuses and placentas from control mothers, which received the vehicle itself, were analyzed.

### Biochemical analysis of maternal blood

All blood samples were collected by retro-orbital bleeding in SST microtainers (Serum Separator Tube, Becton, Dickinson and Company, USA), from animals anesthetized with a drop of local anesthetic (Novesina, Novartis Pharma S.p.A., Italy). All samples were centrifuged in a microcentrifuge (5415R model, Eppendorf s.r.l., Italy) at 13,000 rpm for 7 min to separate the serum. Serum levels of aspartate aminotransferase, alanine aminotransferase, creatinine, blood urea nitrogen and lactate dehydrogenase were measured using the automatic analyzer Keylab (BPC BioSed s.r.l., Rome, Italy).

### Histochemical and immuno-histochemical analysis of maternal tissues

For histochemical analysis, tissues from all animals used in this study were collected, and processed for paraffin embedding. At least twenty sections (one every other 10) of each paraffin block have been routinely stained by H&E. Based on results of the H&E staining, selected paraffin blocks were used for immuno-histochemical analysis. Five micron sections were collected on slides and a part was stained with hematoxylin and eosin, a part used for immuno-histochemical analysis with the rat monoclonal antibody anti-CD31 (clone Mec13.3, BD Pharmingen, NJ, USA). Briefly, slides were de-paraffinized in xylene, rehydrated through the ethanol series and treated in 0.3% H_2_O_2_-methanol for 30 min at room temperature (RT) to block endogenous peroxidase activity. Following a 30 min pre-treatment at 37°C with 30 μg/ml proteinase K (in 0.2 M Tris–HCl, pH 7.2), each section was incubated with a blocking reagent (0.5%, TSA-Indirect Kit, NEN Life Sciences) for 30 min at RT, and finally incubated overnight at 4°C with the anti-CD31 antibody at a concentration of 2.5 μg/ml. For control slides the primary antibody was replaced by a non-specific rat IgG at the same concentration as the primary antibody. After 30 min incubation with secondary biotinylated anti-rat antibodies, staining was revealed using the Tyramide Amplification System (TSA-Indirect Kit, NEN Life Sciences). Slides were counterstained with Mayer’s hematoxylin for 5 min, dehydrated and mounted in Permount mounting medium.

Apoptosis was evaluated with the *In Situ* Cell Death Detection Kit from Roche (Roche Applied Science, IN, USA), following the manufacturer specifications.

### Statistical analysis

When not otherwise stated, data are presented as mean ± standard error. A two-tailed value of P < 0.05 was considered statistically significant. Student’s *t* test was used for inter-group comparison of continuous variables (e.g. weight of dams and fetuses, number of resorptions, fetal size), whereas Fisher’s exact text was used for comparison of categorical variables (e.g. prevalence of dams with at least one malformed embryo, prevalence of malformed embryos, prevalence of liver damage). Analyses were performed by means of the software SPSS Statistics 19 (IBM Corporation, Armonk, NY).

## Competing interests

The authors declare that they have no competing interest.

## Authors’ contributions

LC designed the experiments, performed the analysis of embryos and adult tissues and wrote the manuscript. MM and LV contributed to the embryo and adult tissues experiments and analysis. GP performed the analysis at the Kodak Image apparatus. RB performed the biochemical analysis of maternal blood. CS and MB prepared and characterized the nanoparticles. AB and AM contributed to the preparation of the manuscript. AP designed the experiments and wrote the manuscript. All authors read and approved the final manuscript.
